# Retraction Note: A comprehensive scrutiny to controlled dipolar interactions to intensify the self-heating efficiency of biopolymer encapsulated Tb doped magnetite nanoparticles

**DOI:** 10.1038/s41598-024-57685-9

**Published:** 2024-03-26

**Authors:** Krishna Priya Hazarika, J. P. Borah

**Affiliations:** Nanomagnetism Group, Department of Physics, National Institute of Technology Nagaland, Dimapur, Nagaland 797103 India

Retraction of: *Scientific Reports* 10.1038/s41598-023-50635-x, published online 03 January 2024

The Editors have retracted this Article. After publication, concerns were raised regarding the data presented in Fig. 2. Specifically,

- The difference plots in Fig. 2A, C, and D appear to be identical to those in Fig. 2A, B, and D in Ref.^1^.
- The calculated curve in Fig. 2B appears to be identical to that in the 2% Gd doped panel of Fig. 1D in Ref.^2^.
- The X-ray diffraction patterns shown in Fig. 2 are similar to those shown in Fig. 2 in Ref.^3^.

Additional concerns were raised regarding repetitive features in the X-ray diffraction pattern curves presented in Fig. 2A, B, and D. The Authors shared the original data; however, an expert peer review of this information identified notable differences between the original data and published images. The Editors therefore no longer have confidence in the presented data.

Krishna Priya Hazarika and J. P. Borah disagree with the retraction.
